# The emergence of SARS-CoV-2 in Europe and North America

**DOI:** 10.1126/science.abc8169

**Published:** 2020-09-10

**Authors:** Michael Worobey, Jonathan Pekar, Brendan B. Larsen, Martha I. Nelson, Verity Hill, Jeffrey B. Joy, Andrew Rambaut, Marc A. Suchard, Joel O. Wertheim, Philippe Lemey

**Affiliations:** 1 Department of Ecology and Evolutionary Biology, University of Arizona, Tucson, AZ 85721, USA.; 2 Bioinformatics and Systems Biology Graduate Program, University of California San Diego, La Jolla, CA 92093, USA.; 3 Department of Biomedical Informatics, University of California San Diego, La Jolla, CA 92093, USA.; 4 Fogarty International Center, National Institutes of Health, Bethesda, MD 20892, USA.; 5 Institute of Evolutionary Biology, University of Edinburgh, King’s Buildings, Edinburgh EH9 3FL, UK.; 6 Department of Medicine, University of British Columbia, Vancouver, BC, Canada.; 7 BC Centre for Excellence in HIV/AIDS, Vancouver, BC, Canada.; 8 Bioinformatics Programme, University of British Columbia, Vancouver, BC, Canada.; 9 Department of Biomathematics, David Geffen School of Medicine, University of California Los Angeles, Los Angeles, CA 90095, USA.; 10 Department of Biostatistics, Fielding School of Public Health, University of California Los Angeles, Los Angeles, CA 90095, USA.; 11 Department of Human Genetics, David Geffen School of Medicine, University of California Los Angeles, Los Angeles, CA 90095, USA.; 12 Department of Medicine, University of California San Diego, La Jolla, CA 92093, USA.; 13 KU Leuven Department of Microbiology, Immunology and Transplantation, Rega Institute, Laboratory of Clinical and Epidemiological Virology, Leuven, Belgium.

## Abstract

Accurate understanding of the global spread of emerging viruses is critical for public health responses and for anticipating and preventing future outbreaks. Here we elucidate when, where, and how the earliest sustained severe acute respiratory syndrome coronavirus 2 (SARS-CoV-2) transmission networks became established in Europe and North America. Our results suggest that rapid early interventions successfully prevented early introductions of the virus from taking hold in Germany and the United States. Other, later introductions of the virus from China to both Italy and Washington state, United States, founded the earliest sustained European and North America transmission networks. Our analyses demonstrate the effectiveness of public health measures in preventing onward transmission and show that intensive testing and contact tracing could have prevented SARS-CoV-2 outbreaks from becoming established in these regions.

## A series of unfortunate events

The history of how severe acute respiratory syndrome coronavirus 2 (SARS-CoV-2) spread around the planet has been far from clear. Several narratives have been propagated by social media and, in some cases, national policies were forged in response. Now that many thousands of virus sequences are available, two studies analyzed some of the key early events in the spread of SARS-CoV-2. Bedford *et al.* found that the virus arrived in Washington state in late January or early February. The viral genome from the first case detected had mutations similar to those found in Chinese samples and rapidly spread and dominated subsequent undetected community transmission. The other viruses detected had origins in Europe. Worobey *et al.* found that early introductions into Germany and the west coast of the United States were extinguished by vigorous public health efforts, but these successes were largely unrecognized. Unfortunately, several major travel events occurred in February, including repatriations from China, with lax public health follow-up. Serial, independent introductions triggered the major outbreaks in the United States and Europe that still hold us in the grip of control measures.

*Science*, this issue p. 571, p. 564

## Teaser

Early SARS-CoV-2 transmission networks in Europe and the United States were extinguished, but later travel introduced more virus that became established.

In late 2019, the emergence of severe acute respiratory syndrome coronavirus 2 (SARS-CoV-2), which causes coronavirus disease 2019 (COVID-19), ignited a pandemic that has been associated with more than 500,000 deaths globally as of July 2020. As the original outbreak in Hubei province, China, spilled into other countries, containment strategies focused on travel restrictions, isolation, and contact tracing. Given the virus’s exponential growth rate, delaying the onset of community transmission by even a few weeks likely bought government officials valuable time to establish diagnostic testing capacity and implement social distancing plans.

Viral genetic sequence data can provide critical information about whether viruses separated by time and space are likely to be epidemiologically linked. Genomic data have suggested differences in the timing, spatial origins, and transmission dynamics of early SARS-CoV-2 outbreaks in multiple North American locations, including Washington state (*1*, *2*); the East Coast of the United States (*3*, *4*); California (*5*); and British Columbia (BC), Canada (*5*, *6*). The first confirmed U.S. case was associated with a virus strain (“WA1”) isolated in Washington state from a traveler who returned from Wuhan, China, on 15 January 2020 (*7*). No onward transmission was detected after extensive follow-up in what appeared to be successful containment of the country’s first known incursion of the virus (*8*). However, subsequent identification of viruses that were genetically similar to WA1—first in Washington, then in Connecticut (*3*), California (*5*), BC (*6*), and elsewhere—raised the possibility that WA1 had actually established chains of cryptic transmission that started on 15 January and went undetected for several weeks (*1*, *2*). If true, this introduction would predate early SARS-CoV-2 community transmission chains documented elsewhere on the continent (*3*–*5*) and would establish the Seattle area as the epicenter of the North American epidemic. Hence, it is necessary to resolve this question to determine where the virus first initiated substantial community outbreaks and whether the earliest coast-to-coast spread of the virus within the United States (*3*) was from west to east or from east to west.

In Europe, the first diagnosed case occurred in an employee of an automobile supplier who visited the company’s headquarters in Bavaria, Germany, from Shanghai, China, on 20 January 2020 (*9*). She had been infected with SARS-CoV-2 in Shanghai (after her parents had visited from Wuhan) (*10*) and transmitted the virus to a German man who tested positive on 27 January (*11*) and whose viral genome (“BavPat1”) was sampled on 28 January (*10*). In total, this outbreak infected 16 employees but was apparently contained through rapid testing and isolation (*9*). Italy’s first major outbreak in Lombardy, which was apparent by ~20 February 2020, was associated with viruses closely related to BavPat1 but of a separate lineage (designated “B.1”), which differs from BavPat (a lineage “B” virus) by just 1 nucleotide in the nearly 30,000-nucleotide genome. A narrative took hold that the virus from Germany had not been contained but had been transmitting undetected for weeks and had been carried to Italy by an infected German (*9*, *12*). In addition to igniting a severe outbreak in Italy, this B.1 lineage subsequently spread widely across Europe and beyond, initiating outbreaks in many countries, including the intense U.S. outbreak in New York City (NYC) (*13*, *14*). Greater clarity about the effectiveness of Germany’s early contact tracing efforts has implications for the feasibility of controlling the virus through nonpharmaceutical interventions.

There are a number of limitations in phylogenetic and spatial inferences drawn from SARS-CoV-2 genomic data. SARS-CoV-2 has a relatively long (~29 kb) positive-sense single-stranded RNA genome that evolves at a rate of <1 × 10^−3^ substitutions per site per year, amounting to ~2 substitutions per genome per month. This rate is slower than that of most RNA viruses, owing to the “proofreading” activity encoded by the nonstructural gene *nsp14* (*15*). Consequently, the entire global population of SARS-CoV-2 through March 2020 differed by only 0 to 12 nucleotide substitutions from the inferred ancestor of the entire pandemic. Transmission clusters tend to be defined by 1 to 3 nucleotide differences across the entire viral genome. Phylogeographic inferences are further confounded by the relatively low availability of genomic sequence data from locations that experienced early outbreaks, including Italy, Iran, and the original epicenter in Hubei. The combination of the relatively slow rate of SARS-CoV-2 evolution, its rapid dissemination within and between locations, and unrepresentative sampling presents risks for serious misinterpretation.

In this study, we investigated fundamental questions about when, where, and how SARS-CoV-2 established itself globally. We developed phylogenetic inferences from multiple sources of information—including airline passenger flow data between potential sources and destinations of viral dispersals early in the pandemic, as well as disease incidence data in Hubei province and other locales that likely affected the probability of infected travelers moving the virus around the globe. By combining a genomic epidemiology approach, which aims to account for the effects of undersampling viral genetic diversity in the epicenter of the pandemic, with consideration of expected evolutionary patterns for a novel pathogen with low diversity, we resolved key questions about how and when the SARS-CoV-2 pandemic unfolded in Europe and North America.

## Emergence of SARS-CoV-2 in the United States

A key turning point in the U.S. outbreak occurred when researchers sequenced the first viral genome recovered from a putative case of community transmission in the United States (“WA2,” sampled in the Seattle area on 24 February 2020), reporting on 29 February that it was similar to WA1, the viral variant from the first-diagnosed COVID-19 patient (*1*). This finding led to the suggestion that WA1 might have established cryptic transmission in Washington state in mid-January (*1*). The researchers did, however, acknowledge the possibility of an independent introduction of WA2 separate from that of WA1. This finding fundamentally altered the picture of the SARS-CoV-2 situation in the United States, playing a decisive role in Washington state’s early adoption of intensive social distancing efforts. This, in turn, appeared to explain Washington state’s relative success in controlling the outbreak, compared with that of states that adopted a delayed approach, such as New York.

**Fig. 1:**
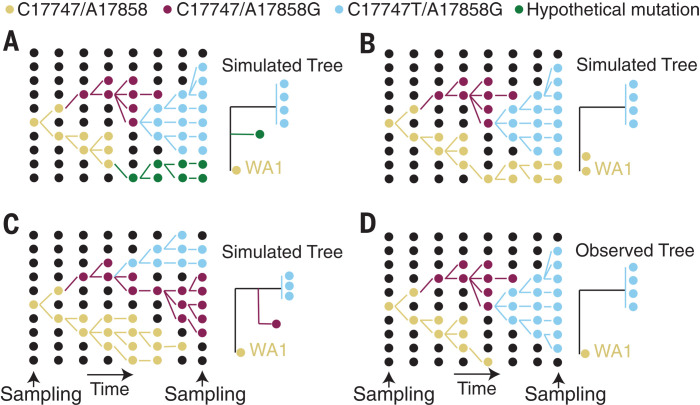
Schematic showing a hypothetical path that the key mutations in the WA outbreak could have taken in a susceptible population, alongside the inferred phylogeny. (**A**) Scenario in which a hypothetical mutation occurs from WA1-like genomes. (**B**) Hypothetical phylogeny in which A17747 and C17858 from the original WA1 virus are maintained in the population and sampled at the end. (**C**) Hypothetical scenario in which a virus that differs from WA1 by one mutation (A17858G) is maintained in the population. (**D**) Observed tree from the WA outbreak.

The availability of hundreds of SARS-CoV-2 genomes sampled in Washington state by mid-March revealed that WA2 belongs to a large, monophyletic clade of “A.1” lineage viruses that accounted for ~85% of cases in Washington state at that point, designated the “Washington state outbreak clade” (*2*) (hereafter “WA outbreak clade”). To investigate whether the WA outbreak clade was initiated in mid-January by WA1, we used these data to simulate the epidemic under the constraint that it had been established by WA1 and then compared the observed evolutionary patterns with those expected under that scenario. A range of phylogenetic patterns could have been observed in this large sample (e.g., Fig. 1, A to C) but were not (Fig. 1D).

To investigate whether the observed pattern of evolution reported in (*1*, *2*) was consistent with the WA outbreak clade having descended from WA1, we used FAVITES (FrAmework for VIral Transmission and Evolution Simulation) to simulate outbreaks (*16*) (fig. S1 and table S1). These simulated outbreaks had a median doubling time of 4.7 days (95% range across simulations: 4.2 to 5.1 days)—including those that originated from so-called “superspreading” events (fig. S2)—and a fixed evolutionary rate of 0.8 × 10^−3^ substitutions per site per year. A duration of 2 months (61 days) was chosen to reflect the time period between WA1 and the implementation of disease mitigation efforts that would affect the median doubling time.

**Fig. 2:**
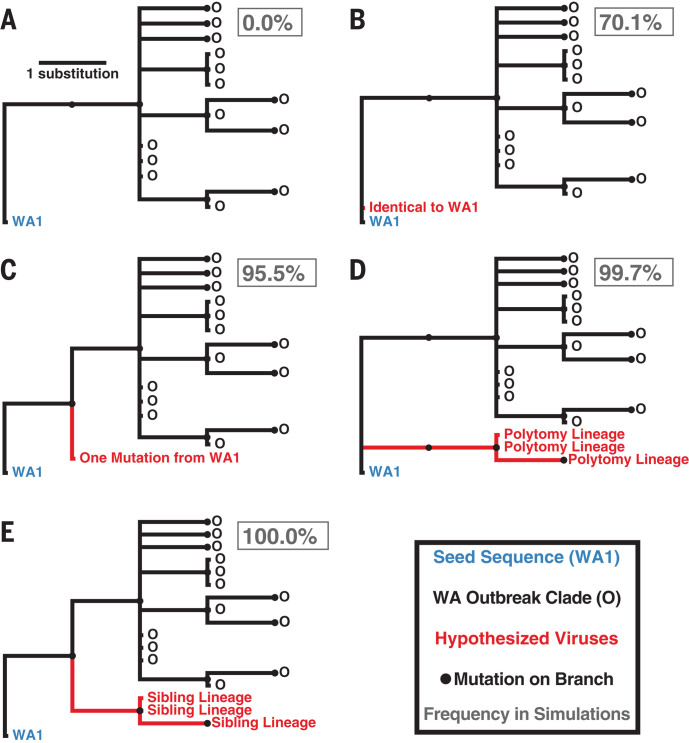
Potential phylogenetic relationships between WA1 and the WA outbreak clade and their occurrence probabilities. (**A**) Observed pattern in which the WA1 genome is the direct ancestor of the outbreak clade, separated by at least two mutations. (**B**) Identical sequence to that of WA1. (**C**) Sequence that diverges from the WA1 sequence by one mutation. (**D**) Lineage forming a basal polytomy with WA1 and the outbreak clade. (**E**) Sibling lineage to the outbreak clade, with fewer than two mutations from WA1 before divergence. The frequency of each relationship across 1000 simulations is reported in the gray box.

We examined the phylogenetic structure of maximum likelihood trees inferred from subsampled simulated viral sequences to determine how frequently they matched the observed relationship between WA1 and the WA outbreak clade. Specifically, a simulation tree matching the observed tree must produce a single branch emanating from WA1 that experiences at least two mutations (C17747T and A17858G in the observed tree) before establishment of a single outbreak clade (Fig. 2A). Alternative patterns include: (i) a virus identical to WA1 (Fig. 2B), (ii) a virus that differs from WA1 by a single mutation (Fig. 2C), (iii) a viral lineage forming a basal polytomy with WA1 and the outbreak clade (Fig. 2D), and (iv) a viral lineage that is a “sibling” of the outbreak clade but experiences fewer than two mutations before divergence (Fig. 2E). The frequency of alternative phylogenetic patterns in the simulated epidemics represents the probability that the true topology (Fig. 2A) could not have occurred if the WA outbreak clade had been initiated by WA1.

In 70.1% of simulations, we observed at least one virus that is genetically identical to WA1, with a median of 12 identical viruses in each simulation (95% range: 0 to 85 identical viruses) (Fig. 2). Not observing a virus identical to WA1 in the real Washington data does not significantly differ from expectation (*P* = 0.299). However, viruses with one mutation from WA1 were observed in 95.5% of simulations, indicating a low probability of failing to detect even a single sequence from Washington within one mutation of WA1 (*P* = 0.045). Lineages forming a basal polytomy with WA1 and the epidemic clade were observed in 99.7% of populations (*P* = 0.003), and 100% of simulations had at least one sibling lineage that diverged before experiencing two mutations and the formation of the outbreak clade (*P* < 0.001). Therefore, even if C17747T and A17858G were linked—a possibility because they are both nonsynonymous mutations located in the nsp13 helicase gene—we would still expect to see descendants of their predecessors in Washington before 15 March. In summary, when we simulated the Washington outbreak beginning with WA1 on 15 January 2020 and sampled 294 genomes in the first two months of this outbreak, we failed to observe a single simulated epidemic that had the characteristics of the real phylogeny (Fig. 2). These findings were robust to simulations that used a slower epidemic doubling time of 5.6 days (95% range: 5.2 to 5.9 days) or an accelerated substitution rate of 1.6 × 10^−3^ substitutions per site per year (*16*) (supplementary text).

Although WA outbreak-related genomes lacking one or the other of the clade-defining substitutions C17747T and A17858G (Fig. 2, C and E) were absent in this initial large sample from Washington state, such genomes have been reported to be very common in nearby BC, Canada (supplementary text). Genomes with the ancestral C17747 state constituted 16 of the first 27 WA outbreak-related genomes sequenced in BC and have been sampled occasionally at much lower frequency in several U.S. states (*3*). Such a high frequency of these viruses in BC but not in Washington state raises the possibility that BC, rather than Washington state, was the site of introduction of the founding virus of this key lineage. Another possibility is that these BC genomes are descendants of a separate A.1 lineage introduction from China. The first scenario seems unlikely because of epidemiological evidence that the outbreak was larger in Washington state than in BC in February and March; the second scenario is unlikely because it would necessitate both introduced lineages to independently acquire the C17747T mutation.

**Fig. 3:**
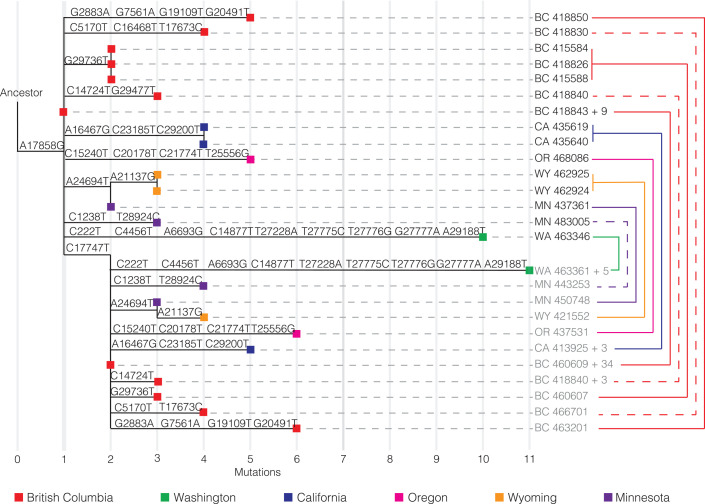
Phylogeny of representative sequences, showing connections between sequences that share derived mutations despite differences at the key site 17747. Derived mutations from ancestral states (relative to the reference sequence hCoV-19/Wuhan/Hu-1/2019|EPI_ISL_402125) are shown above each branch, with position numbers indicated. Branches are connected to taxon names with horizontal dotted lines. The taxon names include a two-letter state or province code, as well as the GISAID accession number. In cases for which more than one sequence is represented, the total number of additional, identical sequences is indicated after the “+” symbol. Sequences that share derived mutations are connected with colored lines on the right, with colors indicating the locations where the connected sequences were sampled. Some lines on the right are dashed for clarity. Names of sequences that contain the derived nucleotide at site 17747 are shaded in gray.

We therefore considered a third hypothesis: that these 16 BC viral genomes contain a sequencing error at position 17747 and, in reality, bear the derived C17747T mutation. We reasoned that if this were the case, some of these genomes might share additional derived mutations with C17747 and A17858G genomes sampled in the same location (i.e., they might be identical or highly similar except for a spurious C17747 base) (supplementary text). As shown in Fig. 3, this is indeed the case: Each of the six C17747 genomes from BC that contain one or more derived mutations at positions other than 17747 and 17858 shared one to four of these mutations with other locally sampled genomes. Such a pattern is virtually impossible to explain through homoplasy events. Observing even one such homoplasy in a genome with more than 29,000 bases is rare; the probability of observing more than one is infinitesimally small. Similarly, the hypothesis that the C17747 state in these genomes is due to multiple, independent T17747C reversions is untenable. Occasional C17747 genomes from California, Oregon, Wyoming, Minnesota, Washington state, and elsewhere also share derived mutations with viruses sampled in the same location (Fig. 3, table S2, and supplementary text). Most of these genomes were generated through the amplicon-based ARTIC protocol, and we speculate that mistaken incorporation of a primer sequence containing C17747 (“nCov2019_58_RIGHT”) may be the cause.

When we investigated an exhaustive collection of genomes sampled in Washington state, including those of viruses sampled after 15 March that are related to the WA outbreak clade (supplementary text), we detected a single virus lineage—“WA-S566,” sampled on 29 March 2020—that lacked the derived C17747T and A17858G mutations found in the rest of the WA outbreak clade. The phylogenetic position of this virus matches the pattern in Fig. 2D, though it differs from WA1 at seven additional sites. Hence, the observed pattern in this larger, and later, sample of ~1000 viral genomes reflects the scenario depicted in Fig. 1A rather than that in Fig. 1D. Consequently, we revisited our WA simulations, sampled 1000 genomes instead of the original 294, and looked for instances in which more than two lineages diverged before the formation of the outbreak clade. In 88.8% of the simulations, we observed two or fewer basally divergent lineages and, therefore, cannot reject a scenario in which WA1 gave rise to only two lineages that diverge as a basal polytomy (*P =* 0.112). However, in 99.0% of simulations, we observed three or more divergent lineages before two mutations (i.e., lineages that experienced zero or one mutation from WA1 before diverging; fig. S3). As a result, it is unlikely that, had it been the ancestral virus, WA1 would have given rise to only the S566 lineage and the WA outbreak clade (*P* = 0.010). Therefore, to explain the presence of S566 and the WA outbreak clade, we must seriously consider the possibility that there were multiple introductions of genetically similar viruses into the United States.

We thus turned to a distinct phylogeographic approach that explicitly considers the relatively late sampling time of WA-S566, along with other temporal, epidemiological, and geographic data. This method accounts for geographical gaps in sampling and integrates relevant covariates for global spatial spread in a Bayesian framework (*16*). We investigated how tree topologies were affected by the inclusion of unsampled viruses assigned to 12 of the most severely undersampled locations, both in China and globally, on the basis of COVID-19 incidence data (*16*). Realistic sampling time distributions were also inferred from COVID-19 incidence data. To better inform placement of unsampled viruses on the phylogeography, we adopted a generalized linear model formulation of the phylogenetic diffusion process (*17*). This approach estimates a significant contribution for both air passenger flow and asymmetric flow in and out of Hubei (both with Bayes factors >8042 and positive log effect sizes; supplementary text).

**Fig. 4:**
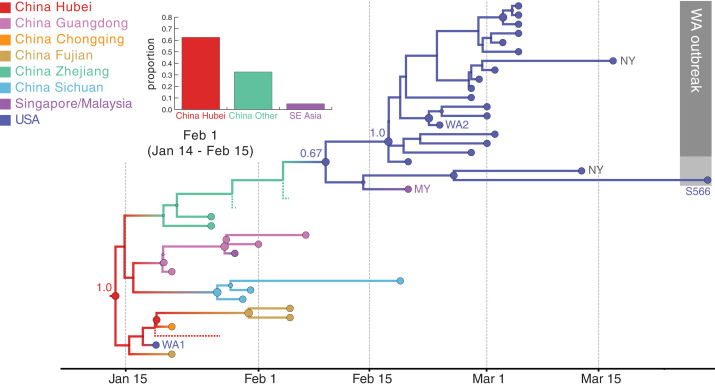
Hypothesis of SARS-CoV-2 entry into Washington state. A subtree of the maximum clade credibility (MCC) tree is shown, depicting the evolutionary relationships inferred between (i) the first identified SARS-CoV-2 case in the United States (WA1); (ii) the clade associated with the Washington state outbreak (including WA2) and related viruses (WA-S566 and a virus from New York); and (iii) closely related viruses that were identified in multiple locations in Asia. Genome sequences sampled at the tips of the phylogeny are represented by circles shaded according to sampling location. Internal node circles, representing posterior clade support values, and branches are shaded similarly by location. Dotted lines represent branches associated with unsampled taxa assigned to Hubei and Zhejiang, China. Posterior location state probabilities are shown for three well-supported key nodes (circle color indicates inferred location state). The inset bar chart summarizes the probability by location for a second introduction giving rise to the WA outbreak clade. The mean date and 95% HPD intervals represent estimated time of introduction from Hubei.

The resulting phylogeny (Fig. 4) provides one reconstruction of the possible evolutionary relationships of WA outbreak viruses and their closest relatives that realistically accounts for major gaps in sequence data. For low-diversity data, a single phylogeny has a resolution that is largely not supported by the full posterior tree distribution containing several plausible phylogeographic scenarios that must be considered, all of which are compatible with the genetic data [e.g., the mutation trees in (*2*) and those available at nextstrain.org]. The posterior maximum clade credibility (MCC) tree (Fig. 4) suggests that the WA outbreak clade (plus S566 and a sibling virus sampled in New York, “NY”) resulted from an introduction from Zhejiang, China, as supported by the clustering of sampled and unsampled taxa from this location. Additionally, although an introduction from a Chinese location other than Hubei yields considerable posterior support (bar chart inset in Fig. 4), Hubei is preferred over Zhejiang for the entire posterior sample as the most likely source for this introduction. Notably, although the genome from NY (near S566 in Fig. 4) is identical to that of WA1, its much more recent sampling time separates it from WA1 (and, similarly, early Chinese sampling) in the time-calibrated phylogeographic reconstruction. The more recent collection date for both this NY sample and S566, as well as modest support [posterior probability (pp) = 0.67] indicating that they share a U.S. location with the WA outbreak viruses, results in a reconstruction with a single introduction for these viruses. Using Markov jump estimates that account for phylogenetic uncertainty (*18*), we inferred 1 February 2020 [95% highest posterior density (HPD): 14 January to 15 February] as the time for this introduction, consistent with the observation that viruses from the WA outbreak clade were likely present during the voyage of the Grand Princess cruise ship to Mexico starting on 11 February (*5*).

**Fig. 5:**
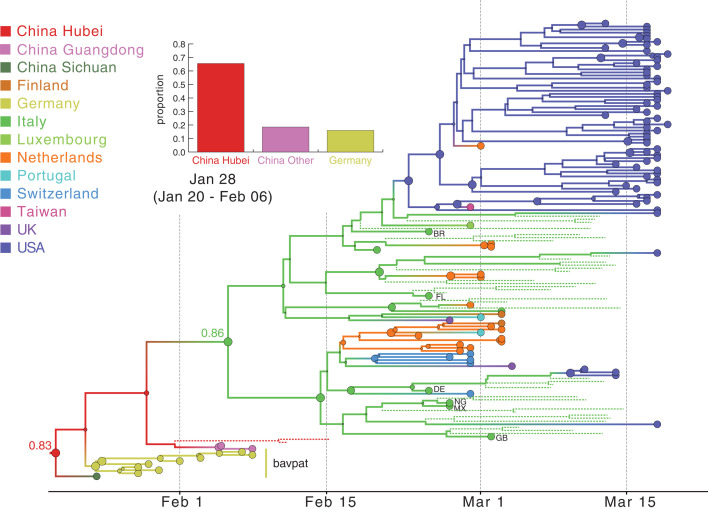
MCC tree of SARS-CoV-2 entry into Europe. A subtree was inferred for viruses from (i) the first outbreak in Europe (Germany, BavPat) and identical viruses from China, (ii) outbreaks in Italy and New York, and (iii) other locations in Europe. Dotted lines represent branches associated with unsampled taxa assigned to Italy and Hubei, China. Country codes are shown at branch tips for genomes sampled from travelers returning from Italy (BR, Brazil; FL, Finland; DE, Germany; NG, Nigeria; MX, Mexico; GB, United Kingdom of Great Britain and Northern Ireland). The inset bar chart summarizes the probability distribution for the location state ancestral to the Italian clade. Other features as described in Fig. 4.

Through a comparison with a time-inhomogeneous model, we show that our estimates are relatively robust to the assumption of constant covariate effect sizes through time (fig. S4). Although the time-inhomogeneous model was fitted to a dataset without unsampled viruses, it also provides strong support for an independent introduction from Hubei (fig. S5). Without unsampled taxa, we estimate a somewhat earlier date for the introduction of the ancestor of the WA outbreak clade plus S566 [26 January 2020 (95% HPD: 15 January to 7 February)], likely because the time-homogeneous analysis allows unsampled taxa from Hubei or other Chinese locations (as in the MCC tree in Fig. 5) to branch off near the WA outbreak clade. In the light of the travel restrictions, specifically those from Hubei, the earlier mean date obtained without unsampled taxa may be the more realistic estimate.

The MCC tree suggests that a Malaysian virus also descended from this introduction (i.e., that it resulted from a subsequent United States–to–Malaysia jump). It is, however, much more plausible that this virus was introduced directly from China to Malaysia, but both the sequence and covariate data in the phylogeographic model lack the information to strongly support this scenario. In light of the simulation results, there is a distinct possibility that S566 and the related NY virus may have descended from a separate introduction from Asia, with the site of arrival in the United States unresolved owing to the presence of both a West Coast and East Coast virus in the clade. Accordingly, an analysis that does not assign a known location to S566 and the related NY virus supports independent introductions from Hubei for these viruses and for the WA outbreak clade (fig. S6), with 7 February (95% HPD: 23 January to 18 February) as the date for the latter.

Consistent with estimates of the introduction date of this viral lineage into Washington state, the Seattle Flu Study tested 6908 archived samples from January and February, of which only 10, from the end of February, were positive (*19*). Our estimates of the introduction date of the WA outbreak clade into Washington state around the end of January or beginning of February 2020 are ~2 weeks later than they would be if the outbreak had originated with WA1’s arrival on 15 January (*2*), implying that: (i) archived “self-swab” samples retrospectively detected the virus within a few weeks of its arrival (*19*), (ii) this Washington state outbreak may have been smaller than estimates based on the assumption of a 15 January arrival of WA1, and (iii) the individual who introduced the founding virus likely arrived in the United States when entry to the country was suspended for non-U.S. residents from China (beginning on 2 February 2020) (*20*), perhaps during the concurrent period when ~40,000 U.S. residents were repatriated from China, with screening described as cursory or lax (*21*). These passengers were directed to a short list of airports, including those in Los Angeles, San Francisco, New York, Chicago, Newark, Detroit, and Seattle (*21*). The late-February timing of COVID-19 cases in Solano County and Santa Clara County in California (*5*) (supplementary text) suggests that self-limited outbreaks may have originated from returning U.S. residents during this period. So although our reconstructions incorporating unsampled lineages do not account for travel restrictions, the remaining influx likely provided an opportunity for a second introduction of virus (distinct from the WA1 lineage), or even multiple such introductions, into Washington state. Recent inferences that there have been >1000 independent introductions of SARS-CoV-2 into the United Kingdom (*22*) lend support to this idea.

## Early establishment of SARS-CoV-2 in Europe

We used a similar approach to investigate whether the Northern Italy SARS-CoV-2 outbreak was introduced from the German outbreak or independently from China: We simulated the Northern Italy outbreak under the hypothetical constraint that it was initiated by a virus imported from the German outbreak (fig. S7) and conducted phylogeographic analyses (Fig. 5). Our simulation framework suggested that the outbreak in Bavaria, Germany, was unlikely to be responsible for initiating the Italian outbreak (see fig. S7 and supplementary results for detailed phylogenetic scenarios). We again used realistic epidemiological parameters to simulate the origins of the Italian outbreak under the assumption that it was associated with viruses genetically related to the German virus BavPat1. Simulations with a median doubling time of 3.4 days (95% range: 2.9 to 4.4 days) resulted in a median epidemic size (including outbreaks that died out) of 725 infections (95% range: 140 to 2847 infections) after 36 days. In the observed phylogeny, the Italian outbreak is the sole descendant lineage from BavPat1. Within the Italian outbreak, no viruses are identical to BavPat1, and 4 of the 27 related viruses included in this analysis are separated from BavPat1 by a single mutation. In simulation, the distributions of identical and one-mutation-divergent viruses are not significantly different from expectation (*P* = 0.156 and 0.157, respectively). However, the lack of at least one descendant lineage that forms a polytomy with BavPat1 and the Italian outbreak significantly differs from expectation (*P* = 0.004). Therefore, it is highly unlikely that BavPat1 or a virus identical to it initiated the Italian outbreak (fig. S7). As with the WA outbreak, these findings were robust to different infection rates and faster evolutionary rates (supplementary text). Notably, therefore, both a WA1-origin of the WA outbreak and a German origin of the Italian outbreak are rejected even by misspecified models of the epidemiological and evolutionary process.

An alternative scenario in which the outbreaks in both Germany and Italy were independently introduced from China is further supported by our phylogeographic inference (Fig. 5). The resulting reconstruction provides stronger support for independent viral introductions from China into Germany and into Italy (pp = 0.84) than for a direct connection between Germany and Italy (pp = 0.16) (Fig. 5). Similar support is obtained for this scenario by a time-inhomogeneous inference without unsampled taxa (fig. S8). These findings emphasize that epidemiological linkages inferred from genetically similar SARS-CoV-2 associated with outbreaks in different locations can be highly tenuous, given low levels of sampled viral genetic diversity and insufficient background data from key locations.

Our approach infers that the European B.1 clade (emanating from the green node labeled 0.86 in Fig. 5), which also dominates in NYC (*14*) and Arizona (*23*), originated in Italy, as might be expected from the epidemiological evidence. Both travel history and unsampled diversity contribute to this inference. Although only two samples in our dataset are from Italy, five additional genomes were obtained from people who arrived from Italy (Fig. 5). The unsampled taxa from Italy further contributed to a reconstruction with stronger support for Italy at the origin of the entire clade (Fig. 5 versus fig. S8; also see fig. S9). The introduction from Hubei to Italy was dated to 28 January 2020 (95% HPD: 20 January to 6 February). This Italian-and-European cluster, in turn, was the source of multiple introductions to NYC (*14*). Using the same approach, we dated the introduction that led to the largest NYC transmission cluster to 12 February 2020 (95% HPD: 3 February to 22 February). This is consistent with the finding that the earliest seropositive samples in NYC were from the week of 17 February through 23 February (*24*).

Hence, even though a second introduction into Washington state (independent of WA1) implies that the Washington transmission cluster had a more recent origin date than under the WA1-origin scenario (~1 February versus 15 January, if it had originated with WA1), the WA outbreak clade still predates the earliest genomically identified transmission clusters elsewhere in the United States: the large one in NYC (*4*) and two smaller, apparently self-limited clusters in California (in Solano County and Santa Clara County) that appear to have been introduced from China (*5*). Of these, the transmission cluster from Santa Clara County appears older, dating to before 22 February 2020 (95% HPD: 5 February to 29 February) (supplementary text).

## Discussion

**Fig. 6:**
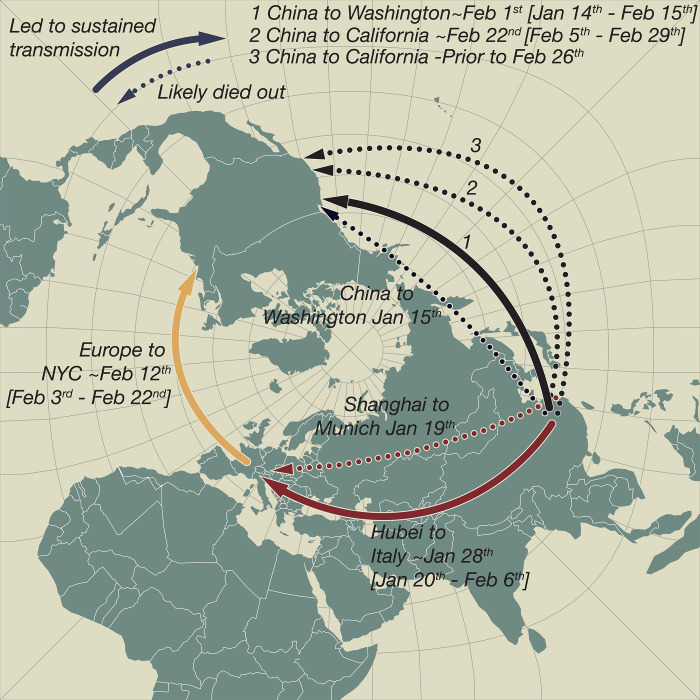
SARS-CoV-2 introductions to Europe and the United States. Pierce projection mapping of early and apparently “dead-end” introductions of SARS-CoV-2 to Europe and the United States. Successful dispersals between late January and mid-February are shown with solid arrows: from Hubei Province, China, to Northern Italy; from China to Washington state; and later from Europe (as the Italian outbreak spread more widely) to NYC and from China to California. Dashed arrows indicate dead-end introductions.

Despite the early successes in containment, SARS-CoV-2 eventually took hold in both Europe and North America during the first 2 months of 2020—first in Italy around the end of January, then in Washington state around the beginning of February, and followed by NYC later that month. Our analyses therefore delineate when widespread community transmission was first established on both continents (Fig. 6) and clarify the period before SARS-CoV-2 establishment when contact tracing and isolation might have been most effective.

Our findings highlight the potential value of establishing intensive, community-level respiratory virus surveillance architectures, such as the Seattle Flu Study, during a pre-pandemic period. The value of detecting cases early, before they have bloomed into an outbreak, cannot be overstated in a pandemic situation (*25*). Given that every delay in case detection reduces the feasibility of containment, it is also worth assessing the impact of lengthy delays in the U.S. Food and Drug Administration’s approval of testing the Seattle Flu Study’s stored samples for SARS-CoV-2.

The public health response to the WA1 case in Washington state and the particularly impressive response to an early outbreak in Germany delayed local COVID-19 outbreaks by a few weeks and bought crucial time for U.S. and European cities, as well as those in other countries, to prepare for the virus when it finally did arrive. Surveillance efforts and genomic analyses subsequently helped close the gap between the onset of sustained community transmission and mitigation measures in Washington state, relative to other locales such as NYC. However, our evidence suggests that the period between the founding of the outbreak and the initiation of mitigation measures in Washington state was not as long as supposed under the WA1-origin hypothesis and that the outbreak may therefore have been smaller than some estimates based on that hypothesis.

Because the evolutionary rate of SARS-CoV-2 is slower than its transmission rate, many identical genomes are rapidly spreading. This genetic similarity places limitations on some inferences, such as calculating the ratio of imported cases to local transmissions in a given area. Nevertheless we have shown that, precisely because of this slow rate, when viral genomes are separated by as few as one mutation, this difference can provide enough information for hypothesis testing when appropriate methods are employed. Bearing this in mind will put us in a better position to understand SARS-CoV-2 in the coming years.

## Notes

**Correction (23 December 2020):** The authors had originally referred readers to an external website that housed the acknowledgments table of the GISAID data used for the study. This table is now included in the supplementary materials as table S3, and the “Data and materials availability” section of the Acknowledgments has been updated. The authors regret the error and thank all GISAID contributors for the use of their data.

## Supplementary Materials

science.sciencemag.org/content/370/6516/564/suppl/DC1 Materials and Methods Supplementary Text Figs. S1 to S9 Tables S1 to S4 References (*27*–*40*) MDAR Reproducibility Checklist
